# Supplementary material for: Are carbon emissions trading and green financial instruments synergistic? -Comprehensive quantitative research based on content analysis
Source: PLoS One. 2024 Mar 7;19(3):e0298601. doi: 10.1371/journal.pone.0298601 (PMC10919601; doi:10.1371/journal.pone.0298601)
Supplement: S1 Data — (ZIP) [file pone.0298601.s001.zip › Data/地级市Z_F工作报告.zip!/╕ú╜¿/╧├├┼╩╨/└┤╫╘╣1⁄2╓┌║┼í╛┬φ┐╦╩2╛▌═°í┐_2006.docx]

各位代表：

　　现在，我代表市人民政府向大会作政府工作报告，请予审议，并请市政协委员和各位列席人员提出意见。

　　一、2005年和“十五”工作回顾

　　2005年，我们在中共厦门市委的领导下，经过全市人民的共同努力，完成了市十二届人大三次会议确定的各项任务，实现了“十五”计划的各项目标。全年生产总值达到1 029亿元，增长16%；工业总产值2 096亿元，增长20.9%；全社会固定资产投资401亿元，增长31.8%；财政总收入209亿元，增长18.9%，其中地方级财政收入103亿元，增长23.2%；城镇居民人均可支配收入16 403元，农民人均纯收入6 230元，分别增长13.6%和10.3%；城镇登记失业率3.79%，居民消费价格指数101，常住人口自然增长率6.25‰；万元生产总值综合能耗0.66吨标准煤，大气质量保持优良；以总分第一名的成绩荣获首批“全国文明城市”。一年来，我们的主要工作是：

　　（一）坚持工业优先，经济保持快速健康发展

　　奠定了工业大发展基础。全年工业投资超过110亿元，高效率建设火炬（翔安）高新技术产业园区、同安工业集中区、软件园二期、机械工业集中区等重点园区，800万平方米工业厂房推出招商。联想移动、柯达图文影像等项目动工建设，戴尔计算机、明达玻璃、太古飞机维修等项目增资扩建，腾龙树脂、ECCO制鞋、钢宇运动器材等一批企业投产。火炬高新区由1平方公里扩大到13.75平方公里，生物与新医药、软件、光电等新兴产业发展迅速，孵化和培育了一批具有自主创新能力的企业，专利申请量增加38.1%。厦华成为我市第二家工业产值超百亿元企业，新增厦工（装载机）、古龙（罐头）、石狮（卷烟）三个“中国驰名商标”和古龙罐头、三圈电池两个“中国名牌产品”。

　　形成了服务业加快发展态势。完成厦门湾八大港区整合，象屿保税物流园区通过中国验收，“区港联动”试点正式运作，海铁联运拓展至江西等地，港口货物吞吐量达到4 770万吨，集装箱吞吐量334万标箱。“十大酒店”、“十大景点”建设顺利推进，举办各类大型展会44个，全年来厦旅游人数突破1 700万人次。引进国美、家乐福、百安居等知名商贸企业，厦门国际银行在上海开设分行，全国首家外资网络公司获准兼业代理保险，法律、会计、咨询等中介服务业进一步向外拓展。第三产业增加值增长15.6%，社会消费品零售总额增长16%，均为近年最快。

　　（二）坚持项目带动，拉开了大开发大建设的序幕

　　突破了征地拆迁瓶颈。在广泛调研的基础上，统筹协调各方利益，出台了全市统一的征地拆迁政策，配套解决被征地农民生产生活出路，有效遏制违法建设，选派干部驻村入户，征地拆迁明显加快，用不到半年的时间完成了过去两年的征地拆迁量，保证了重点项目落地。同时集约使用土地，用地紧张局面有所缓解。

　　创新了项目落实机制。简化审批内容和环节，实行重大项目现场审批或无偿代办，缩短项目前期准备时间，定期召开重点项目调度会，及时解决建设过程中出现的各种问题。建立项目督办制度，专人跟踪，月月督办，即时反馈，确保了重点工程建设进度和质量。

　　加快了项目建设进度。集美大道（国道319—鹰厦铁路立交段）、大嶝大桥建成通车，疏港路改造、环东海域旅游道路、同安湾大桥建设进度加快，翔安海底隧道、成功大道、福厦铁路开工建设，厦漳跨海大桥、厦门—安溪高速公路、龙厦铁路、深厦铁路等项目前期工作进展顺利。海沧港区4、5号码头、东渡港区20号泊位、国际旅游客运码头基本建成。开工建设海沧500千伏变电站，电力进岛第三通道竣工。

　　（三）坚持城乡协调，农村工作取得新成效

　　农民收入大幅度增长。23家市级农业产业化龙头企业产值突破100亿元，带动本市订单农业14.6万亩，本地农户从中获得收入10.1亿元。新建50个现代农业示范基地和无公害农业基地。培训农村富余劳动力9 200人，发放农户联保贷款2.2亿元，实现向非农产业转移2.87万人。1.8万被征地农民参加基本养老保险，新型农村合作医疗保险参保率达93.9%，农村低保标准进一步提高，全市农村低保对象1.5万人做到应保尽保，农民人均纯收入和社会保障水平的提高都是近年来最好的。

　　农村生产生活条件持续改善。建成防汛抗旱指挥中心和高崎闽台避风港，加固海堤4.2公里，完善了一批水利设施，农业防灾减灾能力增强，防御了6次强台风带来的危害。实行“三证四关”，有效防控高致病性禽流感等动物疫病。完成92个农村义务教育和公共卫生项目，整合56所农村学校，改厕500座。通行政村道路实现硬化，行政村客运通达率81%。农村自来水入户率75%，有线电视入户率52%，开通了覆盖12万户农户的农村信息服务网络。与工业区开发相配套，实施“金包银”工程，旧村改造和环境整治取得新进展。

　　（四）坚持外引内联，对外经贸提高到新水平

　　引进外资创近年新高。第九届“投洽会”、第六届世界同安联谊会进一步凸显国际性、实效性，落实了项目对接和跟踪协调制度，扩大了投资促进的成果。全年合同利用外资12.9亿美元，实际利用外资7.1亿美元，投资总额千万美元以上项目74项，外资来源更加广泛，产业链进一步延伸，研发、物流、营销中心等三产引资卓有成效，为新一轮跨越式发展培植了新的经济增长点。

　　外贸口岸地位更加巩固。鼓励骨干企业开拓国际市场，加大扶持中小外贸企业，足额办理出口退税90亿元，提高出口信用保险保费补贴比例，帮助应对技术性壁垒、反倾销等贸易摩擦，东渡港区、机场实施每周七天工作制，口岸物流信息平台辐射到漳州、泉州、龙岩。外贸进出口总额285亿美元，增长18.7%，其中出口172亿美元，增长23.9%，出口额占全省一半以上。

　　对台工作取得新突破。引进台资项目91个，友达光电、长庚医院、客服科技等著名企业落户厦门。吸引台湾企业参加“台交会”、“食品展销会”，扩大对金门的副食品供应，对台贸易总额21.3亿美元。成立海峡两岸农业交流协会，建设台湾农产品在大陆中转销售平台。成功举办海峡两岸旅游博览会、图书交易会、大学校园歌手赛、南音展演暨民间艺术节。厦金航线全年出入境旅客突破50万人次，顺利完成两岸春节包机直航。

　　区域协作有新进展。在城市规划、基础设施、产业布局、旅游线路等方面密切与周边地区的合作，易通卡小额支付系统实现跨行政区域共享，与龙岩、漳州建立粮食、畜禽等农产品产供销合作关系。积极组织国内招商，引进内资项目2 049个，合同利用内资290亿元，实际利用内资115亿元。推进与龙岩、漳州的山海协作，支持南平、三明的抗洪救灾，较好完成了与西藏、宁夏、重庆、新疆的对口帮扶任务。

　　（五）坚持以人为本，执政为民宗旨得到更好体现

　　城市环境进一步改善。集中力量整治市容环境，取缔“黑网吧”，城乡结合部、铁路沿线环境卫生得到有效改善，清理整顿违规排污企业，清淤疏浚筼筜湖，举办国际海洋论坛并形成《厦门宣言》，城市环境综合整治定量考核居全国前列。实施LED（发光二极管）夜景工程，新增绿地357公顷，完成林相改造1.2万亩，造林绿化1.8万亩，开放东坪山市花公园和海湾公园，建成20个街头景观雕塑，更新投放1 000辆出租车，市民和法人基础数据库扩大共享应用，地下管线管理信息系统投入运行，市民生活环境质量继续改善。

　　社会事业全面发展。厦门理工学院一期、集美大学诚毅学院竣工，完成双十、一中、六中的高中部扩建，城乡中、小学办学条件普遍得到改善，在一级达标校就读的高中生占普高总生数40%。城市职业学院完成组建，区级职教资源实现整合。中医院迁建、第一医院和中山医院综合病房大楼等工程进展顺利，优质医疗卫生资源加快向岛外扩展，二、三级医院服务向社区延伸，农村药品供应网络基本建成。成功举办“纪念抗日战争暨世界反法西斯战争胜利60周年全国合唱展演”等文艺活动，地方戏剧首次获得“梅花奖”。国际马拉松赛、第十七届市运会带动了全民健身运动，厦门足球队荣获中甲冠军并晋级中超。

　　社会更加和谐。全年新增就业15.5万人，解决毕业生就业2.2万人。事业单位纳入基本养老保险，吸收建筑和高危行业农民工参加工伤保险，正式发行社会保障卡。深入创建文明社区、和谐劳动关系工业园区。抑制房价过快增长，清理改造现有公房，完善住房公积金和廉租房制度。重视解决群众看病难、看病贵问题，实现平均个人门诊、住院医疗费用零增长。实施医疗救助办法和农村计划生育家庭奖励扶助制度，补助农村贫困生就读中等职业学校，取消进城农民工子女借读费。丰富进城务工人员文化生活，提供法律、培训、娱乐和婚庆服务，免费放映近千场电影。市政府及其部门领导轮值接听市长专线电话，认真处理来信来访，妥善解决了部分群众反映的热点难点问题。关心照顾困难群体，解决了700多户困难家庭的住房问题，博爱超市、爱心超市延伸到农村，慈善捐助蔚然成风。深入开展“双拥”和军警民共建活动，稳妥解决了1999年以前退役士兵安置遗留问题，六个区全部被评为省级双拥模范区。老龄、残疾人、妇女儿童、民族宗教、外事侨务、人防海防、气象地震等工作都取得新的成绩。

　　“平安厦门”深入推进。健全社区、单位、行业治安防控网，严厉打击涉黑涉枪、涉爆涉毒等突出犯罪，大案要案侦破率显著提高，被评为全国社会治安综合治理优秀城市，人民群众对社会治安的满意率达97.75%。“四五”普法工作全面完成。全部镇（街）、村（居）建立调解委员会。继续推进放心食品工程，加大农药残留检测，治理餐桌污染，健全霍乱、人禽流感等疫病防控体系。完善自然灾害和突发事件应急处理预案，加大设备更新投入，建立重大危险源数据库，定期排查和整改安全隐患，专项整治道路交通、危险化学品、非煤矿山和特种设备，各类事故死亡人数下降27.5％。

　　（六）坚持改革创新，政府行政能力进一步提高

　　体制改革不断深化。进一步贯彻《行政许可法》，公布保留的审批事项，取得省政府下放审批事权42项。改革同安电力体制，实现同网同价。组建国有资产管理委员会，“厦门港务”在香港成功上市。实行出租车经营主体重组，公交运营与场站实现分离，完成贷款道路桥梁收费改革。对户外广告、加油站、燃气供应站的经营权进行拍卖，对公交线路和出租车经营、公共绿化和道路养护等实行招标，社会公共资源市场化运作水平进一步提高。

　　勤政廉政工作常抓不懈。完成第一次经济普查工作，深入研究事关长远和国计民生的重大课题，精心制定了“十一五”规划。认真落实市委的各项决策，狠抓高质量的工程建设、高质量的项目招商、高质量的政府服务，进一步转变工作作风，面向企业、面向基层、面向群众，深入实地解决问题。全面推行绩效评估和绩效考评，加大督查督办力度，实施行政过错责任追究制，已确定、已承诺的事项得到较好落实。坚持和完善工程建设招投标、经营性土地招拍挂、产权交易和政府采购“四项制度”，从源头上预防腐败的工作取得新进展。深化部门预算改革，推进国库集中支付，削减一般性开支，行政经费连续两年压缩5%。加强审计监督，查处和纠正违规金额6.3亿元。

　　民主法制建设继续推进。自觉接受人大法律监督、工作监督和政协民主监督，落实定期汇报、专项汇报制度，认真办理人大代表议案、建议292件，政协提案488件，办结率均达100%，满意率分别达93.3%和95.5%。加强同民主党派、工商联和人民团体的联系，诚恳听取各界人士的建议意见。完善政府立法工作，出台和修订了五件规章，公务员学法用法的自觉性增强。推进政务公开，完善政府新闻发布制度，政府网站点击数新增300万次，成为反映社情民意的重要渠道。

　　各位代表！2005年的工作为“十五”计划画上了圆满的句号。过去的五年，是厦门不平凡的五年，是振奋精神、再塑形象的五年；是优化本岛、拓展海湾的五年；是夯实基础、积蓄后劲的五年；是改善民生、和谐发展的五年。

　　五年来，综合经济实力全面提升。生产总值年均增长15.3%，突破1 000亿元，工业总产值突破2 000亿元，财政总收入突破200亿元，这三个关键性指标都比2000年翻了一番。全市225万常住人口人均生产总值超过 5 600美元，财政收入占生产总值比重超过20%，质量和效益保持全国领先。产业整体素质得到提升，重点高新技术企业工业产值占规模以上工业总产值55.3%，产值上亿元企业超过200家，形成了视听通讯、钨材料、半导体照明等一批国家级产业基地。现代服务业发展加快，物流业、旅游业成为新兴支柱产业。城市综合竞争力连年跃升，位居全国200个大中城市第9位。

　　五年来，中心城市建设迅速铺开。累计完成固定资产投资1 355亿元，超过创建经济特区以来前20年的总和。一批城市主干道路、桥梁、隧道建成通车，海港、空港、信息港重大基础设施更加完善，初步形成便捷通畅的海陆空对外辐射通道、连接周边的区域合作通道和城市内部的快速通道。工业按门类加快向岛外聚集，形成新的产业园区。城市规划覆盖全市，城区建设加快向岛外拓展，城市建成区由80平方公里扩大到132平方公里。

　　五年来，改革开放迸发新的活力。调整行政区划，实施简政放权，优化政府流程，激发了各区加快发展的活力。改革事业单位120家，改制国有企业350家，盘活企业资产45亿元，转岗再就业4.1万人。教育、卫生资源整合取得重大进展，经营性社会事业、投融资体制改革有较大突破，市场化水平提高。五年实际利用外资63亿美元（按可比口径计算），实际利用内资314亿元。外贸年进出口总额由100亿美元增长到285亿美元。海港集装箱年吞吐量实现从100万到300万标箱的跨越，位居全国第7位、全球第24强。空港年旅客吞吐量突破600万人次，成为全国干线机场。新增国际友城3个，泰国在厦门设立领事馆，接待外宾团组4 000多批，国际交往进一步扩大。

　　五年来，对台区位优势更加凸显。积极承接台湾光电、石化和现代服务业等产业转移，合同利用台资13.2亿美元，对台贸易额82.9亿美元。推进两岸试点直航，开通福建居民经厦门赴金门旅游，成为两岸春节客运直航包机航点，获准受理五年有效《台湾居民来往大陆通行证》，通过厦金航线往来两岸的台胞累计116万人次。支持台商协会工作，发挥在厦台胞的作用。全面开展与台湾的教育、文化、卫生、体育、人才交流合作，开通厦门卫视、闽南之声广播，厦门作为对台交流合作基地的作用更加突出，为促进祖国统一大业作出了新的贡献。

　　五年来，人民生活品质不断提高。“十五”时期是社会事业和民生保障投入最大、城乡居民受益最多的五年。累计投入111亿元，新建扩建了一大批社会事业项目，厦门爱乐乐团、国际马拉松等品牌影响逐年扩大，厦门正迈向闽西南教育、医疗和文化艺术中心。城乡社会保障水平领先全国，社会治安满意率全省第一。城镇居民人均可支配收入由10 497元增加到16 403元，农民人均纯收入由4 030元增加到6 230元，恩格尔系数由41.9%下降为37％。厦门以文明、温馨、宜居著称，获得“联合国人居奖”和“全国文明城市”两项殊荣，成为公众向往的城市。

　　各位代表！厦门的发展，有目共睹；发展的成果，全民共享。成绩的取得，是在党中央、国务院和福建省委、省政府及厦门市委的正确领导下，在各级人大及各位代表的依法监督和有力支持下，在各级政协及各位委员的民主监督和积极参与下，全市人民齐心协力、艰苦奋斗的结果。在此，我谨代表市人民政府，向全市人民，向各民主党派和工商联、人民团体、各界爱国人士，向离退休的老同志，向中央和省驻厦单位，向驻厦人民解放军、武警官兵和公安干警，向来厦的所有投资者和外来劳动者，向关心支持厦门的港澳同胞、台湾同胞、海外侨胞和国际友人，表示衷心的感谢和崇高的敬意！

　　各位代表，面对新一轮跨越式发展，政府工作还存在一些亟待解决的问题，主要是：社会管理还存在薄弱环节，一些部门回避矛盾、推诿扯皮的现象依然存在，部分政府工作人员积极进取、主动负责的精神不足，缺乏狠抓落实的韧劲。同时，还面临着一些制约经济社会发展的难题，主要是：经济总量偏小，经济腹地、市场空间拓展不够，中心城市的辐射带动功能还较弱；集约用地需要进一步加强；自主创新能力不足，创新型人才和实用型技工偏少；改革的任务还很艰巨，一些体制性障碍亟待破解；人口分布不合理，岛内人口快速增加，交通、就学、就医及环境承载的压力越来越大；被征地农民向二、三产业转移的难度加大，农民增收任务艰巨。对这些问题，我们要有清醒的认识，虚心学习其他城市的先进经验，认真听取广大市民的建议意见，努力加以解决。

　　二、“十一五”时期的展望

　　《厦门市国民经济和社会发展第十一个五年规划纲要（草案）》已经制定，请各位代表予以审议，请政协委员提出意见。在实施过程中，我们必须把握的基本思路是：以科学发展观统领经济社会发展全局，突出扩大经济规模和提高经济效益，突出增强自主创新能力，突出推进海峡西岸中心城市建设，突出经济特区先行先试，突出发挥对台作用，全面构建和谐社会。各位代表！面对中央赋予经济特区新的光荣使命，面对“十一五”发展的宏伟蓝图，我们要认真落实胡锦涛总书记视察福建、厦门的讲话精神，紧紧抓住国家继续鼓励东部地区率先发展、支持海峡西岸经济发展的重大历史机遇，不断推出新举措，持续增创新优势，各方面工作继续走在全省全国的前列。力争经过五年的奋斗，实现三个新跨越。

　　（一）综合经济实力实现新跨越

　　坚持把加快发展作为第一要务，优先发展工业，通过工业带动第三产业，优化第一产业。经济总量比2005年再翻一番，生产总值超过2 000亿元，年均递增15%以上；常住人口人均生产总值力争逼近10 000美元，接近中等发达国家水平。经济效益继续位居全国前列，财政总收入450亿元，年均递增16.5%以上，综合能耗保持国内领先水平。形成海峡西岸先进制造业基地，培育一批具有自主知识产权、国际竞争力较强的优势产业和企业，拥有几个产值上千亿元的产业集群，重点高新技术企业工业产值占规模以上工业产值比重由55.3%提高到68%，科技成为推动经济增长的主要动力。形成辐射周边的现代服务业基地，金融商贸、旅游会展、航运物流业比较繁荣，咨询、会计、评估等中介服务业比较发达，现代服务业对经济增长的推动作用明显增强。

　　（二）中心城市建设实现新跨越

　　围绕“一主四辅八片”的城市空间发展格局，岛内提档次、岛外扩规模，坚持“以港立市”战略，加快海港、空港、信息港建设，增强城市的承载和辐射带动能力。建成翔安海底隧道、成功大道等城市快速通道，形成便捷化、智能化立体交通网；建成福厦铁路、厦深铁路、龙厦铁路，联通国家道路交通主干网，成为中国东南沿海重要交通枢纽。完成海沧、嵩屿港区及配套航道建设，厦门港货物吞吐能力达到1.2亿吨，集装箱吞吐能力超过800万标箱，基本建成国际航运枢纽港。建设高崎机场三期工程，形成1 500万人次的旅客吞吐能力，成为我国重要干线机场。完善互联网基础设施，建成市民服务信息系统、公共信息资源数据库、电子政务公共服务信息平台。基本建成海沧新城、翔安新城、杏北新城，扩大同安、集美城区，岛外形成100平方公里设施完善、环境优美的新城区，城市建成区面积扩大到230平方公里，城市化率提高到75%以上，构成大城区框架。初步形成海峡西岸航运物流、旅游会展、商务营运、金融保险、文化教育、医疗卫生中心，城市国际化、信息化水平显著提高，经济特区先行先试作用更加突出，示范带动效应更加明显。

　　（三）构建和谐社会实现新跨越

　　按照“民主法治、公平正义、诚信友爱、充满活力、安定有序、人与自然和谐相处”的要求，扎实推进社会主义和谐社会建设。率先跨入宽裕型小康社会，城镇居民人均可支配收入和农民人均纯收入分别达到2.5万元和1万元左右，全面普及高中阶段教育，每千人医生数由2.08个上升到3.2个，城乡困难群众皆老有所养、病有所医，低保和社保水平稳居全国前列。人居环境保持全国领先，大气质量保持优良水平，城市建成区绿化覆盖率42%、生活垃圾无害化处理率95%，环境保护和防灾减灾能力进一步提高，资源节约型、环境友好型城市建设取得明显成效。以“生产发展、生活宽裕、乡风文明、村容整洁、管理民主”为目标，建设社会主义新农村，缩小城乡差距，促进城乡和谐。巩固扩大文明城市创建成果，当好全国精神文明建设的排头兵。

　　三、2006年工作建议

　　2006年，是实施“十一五”规划的开局之年。主要预期目标为：生产总值增长16%，地方级财政收入增长23%，城镇居民人均可支配收入和农民人均纯收入均增长9%，城镇登记失业率控制在4%以内，居民消费价格指数102左右，人口出生率控制在10.8‰以内。在实际工作中要加倍努力，力争完成得更好。政府主要工作是：

　　（一）加快发展先进制造业，在结构调整中提升规模效益

　　扩大工业集聚规模。加快火炬(翔安)高新技术产业园区、同安工业集中区、软件园二期、机械工业集中区建设，完善园区配套，吸引企业入驻园区，争取一批大型骨干项目开工投产、早见效益。鼓励现有企业提升改造、增资扩产，进一步延伸产业链，做大工业规模。推动联想移动、明达光电、趋动科技等项目竣工投产，抓好友达光电、华映工业园、太古飞机维修五期项目建设和厦华易地搬迁改造，力争开工建设PX（对二甲苯）项目，推进PTA（精对苯甲酸）等重大项目的前期工作，确保全年实现工业总产值2 500亿元。

　　增强自主创新能力。加快构建企业为主体的区域创新体系，增强集成创新和引进消化吸收再创新能力，不断提高科技进步对经济增长的贡献率。增加财政拨款1亿元，重点扶持拥有自主知识产权的重大产业科技项目。引导企业、高校、科研机构建立技术创新联盟，促进产学研合作，重点建设科技创业广场、厦大“国家大学科技园”等孵化基地。支持企业建立研发机构，加快建设诊断试剂、疫苗工程技术研究中心和产业基地，建设半导体照明测试中心，争取钨材料、汽车工程等一批企业研发机构升级。实施名牌带动战略，加强知识产权保护，鼓励开发专利和品牌，推动由“厦门制造”向“厦门创造”的转变。

　　大力发展循环经济。落实市人大常委会《关于发展循环经济的决定》，制定相关政策措施。倡导节能节材节水，集约使用土地和海域，推行清洁生产，推进可再生资源回收利用。加快建设鼓浪屿循环经济实验区，完成建设部节能试点示范和美国能源基金合作项目，逐步推广中水回用，鼓励用水器具节约型改造，推广使用新型墙体材料。普及循环经济知识，引导绿色消费，努力创建节约型社会。

　　（二）大力拓展内需市场，提升中心城市服务功能

　　积极扩大消费拉动。抓住中央实施扩大内需政策的有利时机，清理抑制消费的不合理规定，做好流通体制改革试点。大力拓展消费领域，加紧建设汽车、名牌时装和港澳台商品等各种消费中心，建设同安建材等大型专业市场，全面启用明发商业广场，精心筹办各类节庆活动，打造会展、旅游、商贸互动的活动平台，促进旅游休闲、海上运动、保健健身、教育培训、演艺文化消费。坚持以解决市民住房为主的调控原则，加大岛外开发建设力度，扩大普通商品房供应量，改革经济适用房和廉租房的供应方式，发展房屋交易和租赁市场，满足消费、抑制投机，保持房价基本稳定。支持大卖场、连锁店向岛外和农村延伸，鼓励企业向省内和中西部拓展市场，提高内销份额。

　　增强城市现代服务功能。争取国际知名航商增辟航线，积极培育内支线和内贸线，拓展海铁联运，建设以厦门为平台的福建电子口岸，推进口岸大通关。发挥保税物流园区作用，发展中转、拼装和分拨业务，吸引周边货物经厦门口岸进出。开发五缘湾、观音山等营运中心区，吸引更多企业来厦经营。抓紧园博园和天竺山森林公园建设，扩建改造万石植物园，建成喜来登等高星级酒店，续建国际会议中心和国际游艇码头，建设旅游集散中心，培育会展运营商，吸引境内外企业来我市办展或设立展览公司，形成有特色、有影响、有规模的旅游会展品牌。加快发展咨询、评估、会计、法律、仲裁、公证等中介服务业。建成城市地理数据库，完善公共服务信息平台，拓展信息服务范围。

　　（三）着力提高农民收入，建设社会主义新农村

　　广辟农民稳定增收渠道。继续贯彻工业反哺农业、城市支持农村的方针，加大财政转移支付力度，巩固农村税费改革成果。帮助农村未升学青年接受职业教育，鼓励企业招用本地农民工，政府提供保洁员、护林员等公益性岗位，确保转移农村劳动力1.2万人以上。加大对农业龙头企业的扶持力度，拓展订单农业，让千家万户农民得到更多实惠。大力发展无公害农业，积极培育有效益有特色的种苗业和生态休闲观光农业，提高种养业收入。加快推进“金包银”工程，统一建设外口公寓、商业服务、住宅小区等配套设施，确保每户被征地农民有住房保障和稳定的生活来源。扶持老区基点村、经济薄弱村发展，推进移民造福工程，抓好重点水源保护区和边远山村整体迁移试点。继续推广被征地农民基本养老保险和大病医疗保险，完善农村困难群体救助制度，努力减轻农民的后顾之忧。

　　积极推进农村城市化。探索建立“政府引导、农民为主、社会参与”的城市化投资机制，通过工业集中区、重大产业项目带动农村城市化。按照异地新建、就地改造、环境整治三种类型，做好20个旧村改造和新村建设试点。推进镇改街、村改居，基本完成岛内村改居后集体资产改制，加强对村财、组财的监督管理。做好农村基层组织换届选举，稳妥实施乡镇机构改革。加强农村文化体育设施建设，力争提前一年完成156个农村义务教育和公共卫生建设项目，实现行政村有线电视入户率70％，继续改水改厕、绿化村庄，基本完成自来水进村入户建设任务。

　　（四）落实项目带动战略，加快城乡基础设施建设

　　建设重点继续向岛外集中。加快土地利用总体规划修编，积极争取核减基本农田总量，确保建设用地需要。做好岛外新城区规划，整治马銮湾区，续建海沧新城、杏北新城和翔安新城。加快建设翔安隧道、福厦铁路，开工建设公铁（杏林）大桥，抓紧海沧港区和嵩屿港区在建项目的进度。新建电力进岛第四通道和五显220千伏变电站，确保海沧500千伏变电站和翔安220千伏变电站投产。新建扩建海沧、翔安、梅山水厂，加快石兜水库扩容，完成九龙江北溪引水渠道改造，保证供水安全。力促LNG（液化天然气）项目动工，加紧集美、海沧管道燃气建设。打通仙岳路东段，加紧建设成功大道和环岛干道，完成疏港路、仙岳路西段改建，加强岛内外的连接。改造中华片区、百家村和厦港旧城区，加快机场三期前期工作。

　　确保工程建设质量。落实工程质量终身负责制，把好工程勘察、设计、施工、监理、验收每一个环节，明确业主法人是工程质量的第一责任人，强调施工单位的主体责任和监理单位的监理责任，决不允许以任何理由降低技术标准和质量要求。积极推广新材料、新技术、新设备，依靠科技提高建设质量和水平。进一步完善工程代建制度，严格执行项目经理和建筑工人持证上岗制度，建立全行业信用档案，对优质工程给予奖励，对违规的单位和个人，严格处理直至清出厦门建筑市场。

　　（五）推进改革开放，保持外向型经济的领先优势

　　集中力量加大招商。以“投洽会”和“台交会”十周年为契机，做好工业集中区、软件园区、营运中心区的项目引进，积极参加“6·18”等项目对接会，努力扩大引资规模。制造业重点引进一批集聚能力强的大项目，带动引进相关联的中小项目，延伸产业链和壮大产业集群。服务业着力引进航运物流、旅游会展、中介服务等新项目，提升城市服务功能。引导国内外企业在厦设立研发中心、销售中心和结算中心，吸引跨国公司将高附加值环节、服务外包业务转移到厦门，支持国家重点企业和大型民营企业在厦投资。

　　提升外贸竞争能力。适应国家外贸政策调整，发挥保税区、出口加工区、区港联动的政策效应，优化出口商品结构，扶持厦华高端彩电等一批自主品牌、工贸结合、高新技术产品出口，增加出口附加值。组织企业出国出境参展，落实出口信用保险保费补贴，支持中小企业开拓国际市场。积极发展旅游、教育、医疗等领域的国际服务贸易。支持金龙汽车等有竞争实力的企业在海外设点，扩大境外加工贸易、工程承包和劳务输出。建立出口商品应对技术性壁垒监测预警系统，合理运用WTO规则、国际通行做法，维护好企业的权益。出台重点商品进口扶持政策，培育电子、工业原辅料等商品进口分拨配送中心。

　　创新体制机制。完善市对区管理体制，进一步发挥区级积极性。全面实施国库集中支付制度，试行财政专项支出预算绩效考评。加强国有资产监管，完善国有资本经营业绩考核体系，整合国有资产投资公司和授权经营集团，深化国企股份制改革。推动市商业银行引进战略投资者，完成农信社改革，稳步推进上市公司股权分置改革，推出有条件的企业在境内外上市融资及后续融资。继续做好事业单位改革，推进行政资源和社会公共资源配置市场化，将公交线路、出租车牌照等竞争性资源推向市场，实行公开招、拍、挂。继续鼓励、支持和引导民营经济发展，进一步创造条件让非公资本进入法律法规未禁止的各个领域。

　　促进国内区域协作。密切厦漳龙山海协作和厦漳泉城市联盟，推进闽西南五市和闽粤赣十三市区域协作，加强区域内城市规划、基础设施、产业布局、环境保护、旅游线路、海洋管理对接，推动教育文化、医疗卫生等领域交流合作，加强与内陆腹地口岸的通关协作，促进资金、技术、人才等生产要素自由流动。认真做好对口支援、对口帮扶工作。

　　（六）依托“五缘”强化“六求”，促进两岸经济文化交流

　　充分发挥与台湾地缘近、血缘亲、文缘深、商缘广、法缘久的优势，力求紧密经贸联系、两岸直接“三通”、旅游双向对接、农业全面合作、文化深入交流和载体平台建设。争取扩大台商投资区，更好承接台湾新一波产业转移，争取在光电、软件和动漫产业等方面取得新突破。进一步开放对台服务贸易，加大力度引进航运物流、金融保险、教育卫生和中介服务业。推进长庚医院等台资项目建设，办好台湾学者创业园。建设厦门（闽台）花卉高科技园、闽台中心渔港，建设好台湾水果、花卉等农副产品进入大陆的集散地。鼓励台湾金融机构入驻，力争率先开办人民币与新台币计价结算业务。拓展厦金旅游，争取开通省外居民赴金门旅游，完善厦金红十字生命救助绿色通道。对台湾、金门学生来厦就读实行同等收费。办好厦门卫视、闽南之声广播、海峡新闻网，发展闽南地方影视、音乐和戏剧文化。增进两岸青少年、妇女以及民间团体的交往，促进两岸民间民俗交流。

　　（七）落实好社会事业建设项目，保持经济社会协调发展

　　加快发展社会事业。加快集美、翔安文教区的项目建设，大力发展职业教育、成人教育，提高高等教育水平。继续高质量普及义务教育，加快农村中心校建设，参评高级职称的城市中小学教师一律到农村支教一年。按期完成第一医院、中山医院、中医院等扩建新建项目，建成医高专、疾控中心、紧急救援中心，分批改造村级卫生所。强化流动人口计生管理和服务，实行免费婚检和免费优生检测，提高出生人口质量。开展“关爱女孩”行动，治理出生人口性别比偏高问题。精心办好第四届世界合唱比赛，办成有影响的国际文化交流活动。深化文化体制改革，重视文物保护工作，提高油画、动漫、收藏品交易、出版印刷业的发展水平，壮大厦门特色文化产业。全面推广数字电视，启用文化艺术中心，开展志书的续编工作。建成嘉庚体育馆、工人体育馆和翔安体育中心，续建国家乒乓球南方训练基地等项目。继续办好国际马拉松赛，推动全民健身。

　　实施人才强市战略。营造人尽其才、才尽其用的良好环境，按照增加总量、提升质量、盘活存量的思路，修订人才政策。结合产业发展、社会需求，建立全民教育培训资助制度，加快培养本地高级人才，重点加大光电子、生物与新医药、软件等新兴产业人才的引进和培养。适应教育、卫生等规模扩张，加大名师、名医等紧缺人才引进力度。加快中青年为主体的企业家群体的培养，更加重视职业经理人、高级技工队伍建设，引导更多优秀人才向重点产业集聚。建立一批高水平实验室和科研机构，打造高精尖人才、学科带头人载体，适应大开发大发展的需要。

　　（八）维护群众利益，不断提高人民群众生活品质

　　落实民生保障措施。加强社区建设，进一步提升社区自治水平和服务功能。扎实做好整治农贸市场、规范小区物业管理、提高公交服务水平等十件为民办实事项目。努力扩大就业和再就业，举办专场招聘会，重点帮扶城镇“零就业”家庭和农村被征地农民等困难群体。鼓励用人单位招收本地生源大中专毕业生，完善技能培训、职业见习制度。修订基本养老保险政策，加强社会保险基金的安全监管。确保农村学生接受义务教育，全部免收学杂费，为贫困家庭学生免费提供教科书、补助寄宿生活费。继续保持医疗费用零增长，努力降低药品加成率，严禁不合理的医疗检查和小病开大处方，做好低收入困难群众的医疗救助，减轻群众医疗负担。增加医保定点药店，强化药品监督，保障用药安全方便。进一步提高蔬菜、肉类等农产品检测率，确保食品安全。引导商场、酒店等公共场所明确卫生间标识，扩大对公众开放。优先发展公共交通，加强停车场建设，提高交通疏导水平。保证燃气、自来水等生活必需品的安全稳定供应。积极创造条件，让进城务工人员享受市民待遇，帮助解决工资待遇、社会保障、子女就学等问题。

　　提升“平安厦门”水平。落实社会治安综合治理责任制，完善治安防控体系，加大社区和路面报警与监控系统建设，深入开展打击“两抢一盗”、“六合彩”和涉毒犯罪等专项行动。实施青少年违法犯罪社区预防计划，加强校园周边治安综合治理。启动“五五”普法工作。进一步完善多元化纠纷解决机制，重视信访工作，加强人民调解，努力把矛盾化解在萌芽状态。严格落实安全生产责任制，深化道路交通、生产施工、危险化学品、非煤矿山等安全专项整治。强化动植物疫病防控措施，建设动物无害化处理场，继续实行“三证四关”，严防高致病性禽流感等疫情。建设突发公共事件应急指挥系统，提高110等应急处置的快速反应能力，加快人防体系建设，确保人民生命和财产安全。继续做好新形势下的双拥工作，关心复转军人，支持驻厦部队、武警部队、民兵预备役部队建设。

　　持续改善人居环境。巩固和深化文明城市创建成果，按照“各负其责、常抓不懈、完善机制、长效管理”要求，抓好日常管理、基层管理和行业管理。继续美化绿化城市，完成1.5万亩林相改造和造林更新，治理裸露山体和水土流失，加快建设香山、大帽山森林公园。突出厦门海洋的生态、航运、旅游三大功能，逐步退出海上养殖，加快海域整治，防堵陆源污染，建设湿地生态公园。综合整治湖边水库，完成筼筜湖排涝及周边截污工程，治理官浔溪、过芸溪、九溪等流域。完成东孚垃圾填埋场扩容，加快城市生活垃圾分类处理厂建设，开工建设固体废弃物处理中心。加强重点污染源环境安全监管，严防各类污染事故发生。

　　四、持之以恒建设对人民高度负责的政府

　　对人民高度负责，是本届政府作出的庄严承诺。我们将持之以恒，忠诚履职，当好公仆，更好地做到权为民所用、情为民所系、利为民所谋。

　　（一）凝聚合力

　　牢固树立机遇意识、发展意识、大局意识、责任意识和忧患意识，最广泛最充分地调动一切积极因素，把上上下下、方方面面的智慧和力量，进一步凝聚到新一轮跨越式发展上来，形成朝气蓬勃、奋发有为的精神风貌，形成团结协作、攻坚克难的强大合力。坚持定期向市人大常委会报告重要工作的制度，完善联系沟通机制；及时向市政协通报重要工作，更好地尊重和发挥人民政协的参政议政职能；认真听取民主党派、工商联、无党派人士和各人民团体的意见，广开市民建言献策渠道。保持高度敬业的态度和敢于碰硬的作风，在其位、谋其政、负其责。配合做好换届工作，保证思想不乱、精力不散、工作不断。以是否促进发展为共识、为标准、为大局，对基层一线的同志，对敢抓敢管的同志，多一些理解、多一些鼓励、多一些支持，真正做到上级为下级撑腰，下级对上级负责，共同为发展用劲。

　　（二）锐意创新

　　坚持经济特区先行先试，以超前的思维想大事、谋大局，努力使改革措施符合实际情况、反映客观规律，得到广大人民群众的拥护和支持，力争在一些关键环节和重点领域实现新突破。推进政府工作管理创新和制度创新，巩固行政审批制度改革成果，取消的行政审批项目，任何单位不得变通或变相恢复。进一步推进电子政务，扩大网上审批范围。继续转变工作作风，提高政府机关效能，为企业、为市民提供更优更多的公共服务。增强按市场经济规律、按国际通行规则领导经济工作的意识和能力，加强对各项改革的统筹协调和综合配套，及时研究改革过程中出现的新情况新问题，营造促进改革、鼓励创新、宽容失败的良好氛围。

　　（三）务求落实

　　抓不抓落实、如何抓落实、能否抓落实，反映党性和觉悟，体现作风和意志，检验能力和水平。要脚踏实地、力戒浮躁，在任务面前勇于承担、在困难面前勇于进取、在矛盾面前勇于开拓，从人民要求做、眼前能够做、自身应该做的事情做起。落实重大部署和决策，必须开动脑筋、结合实际、深入贯彻；落实已经开始做的事项，必须加快进度、确保质量、务求实效；落实“十一五”规划确定的任务，必须围绕项目、破解难题、积极推进。实行严格的目标责任管理，务必细化任务、分解到人，抓好跟踪督办、检查反馈。认真办理人大代表议案、建议和政协提案，加强面对面沟通，确保办结率，提高满意率。坚持少说多做、说到做到、说好做好，确保说过的话能算数、承诺的事能兑现。

　　（四）廉洁高效

　　落实《公务员法》，贯彻国务院《全面推进依法行政实施纲要》，强化行政执法责任，加强公务员队伍建设。抓紧建立健全教育、制度、监督并重的惩治和预防腐败体系，强化对权力的制约监督，形成反腐倡廉长效机制，积极预防职务犯罪，保证领导干部廉洁从政。以公权力大、公益性强、公众关注程度高的部门为重点，强化对财政资金使用、国有资产运营、工程建设招投标、土地使用权出让、行政审批权运用等方面的监督。认真开展商业贿赂专项治理，严肃查处公务员插手商业活动收受贿赂的案件。支持监察、审计部门依法独立履行监督职责，做好领导干部任期经济责任审计。厉行节约、勤俭办事，严格控制一般性行政开支。政府机关工作人员特别是领导干部，要精于业务、勤于动脑、善于出活，树立推动工作、为民负责、公正廉洁的形象。加快工作节奏，提高工作效率，大力倡导开短会、讲短话、发短文，集中精力谋大事、抓急事、解难事。深入基层察民情、知民意、解民忧，着力解决群众最关心、最直接、最现实的利益问题。各位代表！“十一五”的蓝图已经绘就，新的征程已经开始。让我们高举邓小平理论伟大旗帜，全面贯彻“三个代表”重要思想，更加紧密地团结在以胡锦涛同志为总书记的党中央周围，在中共厦门市委的坚强领导下，锐意进取，开拓创新，为推进新一轮跨越式发展而努力奋斗！
